# Oral mucosa immunity: ultimate strategy to stop spreading of pandemic viruses

**DOI:** 10.3389/fimmu.2023.1220610

**Published:** 2023-10-19

**Authors:** Hyesun Jang, Michele Matsuoka, Marcelo Freire

**Affiliations:** ^1^ Genomic Medicine and Infectious Diseases, J. Craig Venter Institute, La Jolla, CA, United States; ^2^ Division of Infectious Diseases and Global Public Health Department of Medicine, University of California San Diego, La Jolla, CA, United States

**Keywords:** oral mucosa, RNA viruses, pandemics, saliva immunity, mucosal vaccine

## Abstract

Global pandemics are most likely initiated via zoonotic transmission to humans in which respiratory viruses infect airways with relevance to mucosal systems. Out of the known pandemics, five were initiated by respiratory viruses including current ongoing coronavirus disease 2019 (COVID-19). Striking progress in vaccine development and therapeutics has helped ameliorate the mortality and morbidity by infectious agents. Yet, organism replication and virus spread through mucosal tissues cannot be directly controlled by parenteral vaccines. A novel mitigation strategy is needed to elicit robust mucosal protection and broadly neutralizing activities to hamper virus entry mechanisms and inhibit transmission. This review focuses on the oral mucosa, which is a critical site of viral transmission and promising target to elicit sterile immunity. In addition to reviewing historic pandemics initiated by the zoonotic respiratory RNA viruses and the oral mucosal tissues, we discuss unique features of the oral immune responses. We address barriers and new prospects related to developing novel therapeutics to elicit protective immunity at the mucosal level to ultimately control transmission.

## 1 Zoonotic respiratory RNA viruses are linked to global pandemics

Pandemic refers to the explosive outbreaks of communicable diseases on a global scale (1–3). The scale, geographic location, and duration of pandemics are unpredictable (4, 5). Historically, the most devastating pandemics were initiated by cross-species transmission of pathogens, such as Justinian plague (541-542 AD), the Black Death (1347-1351), flu pandemics (Spanish flu in 1918, Asian flu in 1957, Hong Kong flu in 1968, Russian flu 1977, Swine flu in 2009), and the ongoing SARS-CoV-2 COVID-19 pandemics (2019-current) (6–9). Since most human populations are immunologically naive, wildlife pathogens that acquired a susceptibility to humans can spread rapidly (10). Still, cross-species transmission from animal to human is not as common and requires successful adaptation to maintain long-term human to human transmission (11–14). Wolfe et al. summarized five progressive stages of animal microbe’s human adaptation: 1) exclusivity to animals; 2) obtaining non-sustainable animal-to-human transmission; 3) limited human-to-human transmission; 4) sustained human-to-human transmission without the need for an intermediate host (influenza A, SARS, MERS, SARS-CoV-2,Vibrio cholerae, and dengue virus); and 5) exclusive circulation in humans (15). As humans encroach into the natural habitats of wildlife and as human population, travel, and trade increases, so does the risk of spillover events (16). Domestic animals serve as intermediate hosts to create novel zoonotic pathogens, increasing the chance of transmission from wildlife (17, 18). Emergence of the pandemic 2009 H1N1 virus (pdm09 H1N1) serves as a prime example where the novel virus was created by a triple genetic reassortment event (influenza genes derived from North American swine, humans, and birds) which most likely occurred in domesticated pigs (19–21). Exceptional mutation rates and short generation times are highly advantageous to RNA viruses, allowing them to adapt to new host systems and break the species barrier by compatibility to host cell receptors, cellular enzyme systems, or tissue tropism (22, 23). Mutation rates of RNA viruses can roughly occur at rates of six orders of magnitude greater than those of their cellular hosts (23). Across multiple studies, a critical part of emerging pathogens (25-44%) in humans is reported to be related to respiratory RNA viruses (24–27).

The global pandemics affecting all five continents almost simultaneously were initiated by zoonotic respiratory RNA viruses including influenza and the coronaviruses. Currently, vaccines are the most efficacious measure to reduce the disease severity and mortality of respiratory viral diseases (28, 29). However, due to the biased immunogenicity to elicit systemic neutralizing antibody response, vaccinations cannot stop the spread of the virus at mucosal surfaces (30–33). Silent spread of viruses among asymptomatic patients can further generate novel escaping mutants (34–37) and impact public health.

## 2 Salivary droplets as transmission source of zoonotic respiratory RNA viruses

Respiratory RNA viruses primarily infect and replicate at respiratory tracts, and the amplified viruses shed their progeny into mucosal droplets, often spread by coughing or sneezing (38, 39). Considering the poor stability of RNA and viral envelope structure, transmission of aerosolized particles had been, historically, less supported (40). Due to this belief, the efficacy of facial masks was questioned in preventing transmission of the respiratory viruses during the initial phase of the COVID-19 pandemic (41). The role of aerosolized particles in transmission of respiratory particles has been more supported as experiencing explosive incidence of the COVID-19 cases in indoor environments that are poorly ventilated, such as meatpacking factories, cruise ships, and churches (40).

For the transmission of highly attenuated SARS-CoV-2 variant strains, salivary droplets generated during speech have been increasingly considered as a major transmission vehicle for the asymptomatic carriers lacking respiratory symptoms (coughing and sneezing) (42, 43). While the SARS-CoV-2 virus is considered a respiratory pathogen, the virus is known to replicate in a variety of tissues and organs expressing the ACE2/TMPRSS receptors, including gingival tissues and salivary glands (44).

This is also consistent with human adapted influenza viruses and oral epithelium. It requires galactose linked to α-2,6-sialic acid, abundantly expressed on epithelial cells of the upper respiratory tract, including oropharynx (45). While avian influenza viruses preferentially bind to the α-2,3-SA expressed in the human lower respiratory tract, human adapted zoonotic influenza viruses replicate in the oropharyngeal airway and shed into the salivary droplets. Responsible for the 2009 pandemic, the A/(H1N1)pdm09 virus has been reported to bind to α-2,6-SA and, to a limited extent, to α-2,3-SA (45, 46). In the case of the highly pathogenic avian influenza virus H5N1 viruses, one of the most devastating candidate pandemic virus strains, can also infect and replicate in cells of the nasopharyngeal and oropharyngeal epithelia (47). Influenza is also known to be detected in saliva (48, 49). A recent study showed no significant difference in detection rate of influenza virus detection rate between saliva and nasopharyngeal swabs (48).

**Figure 1:**
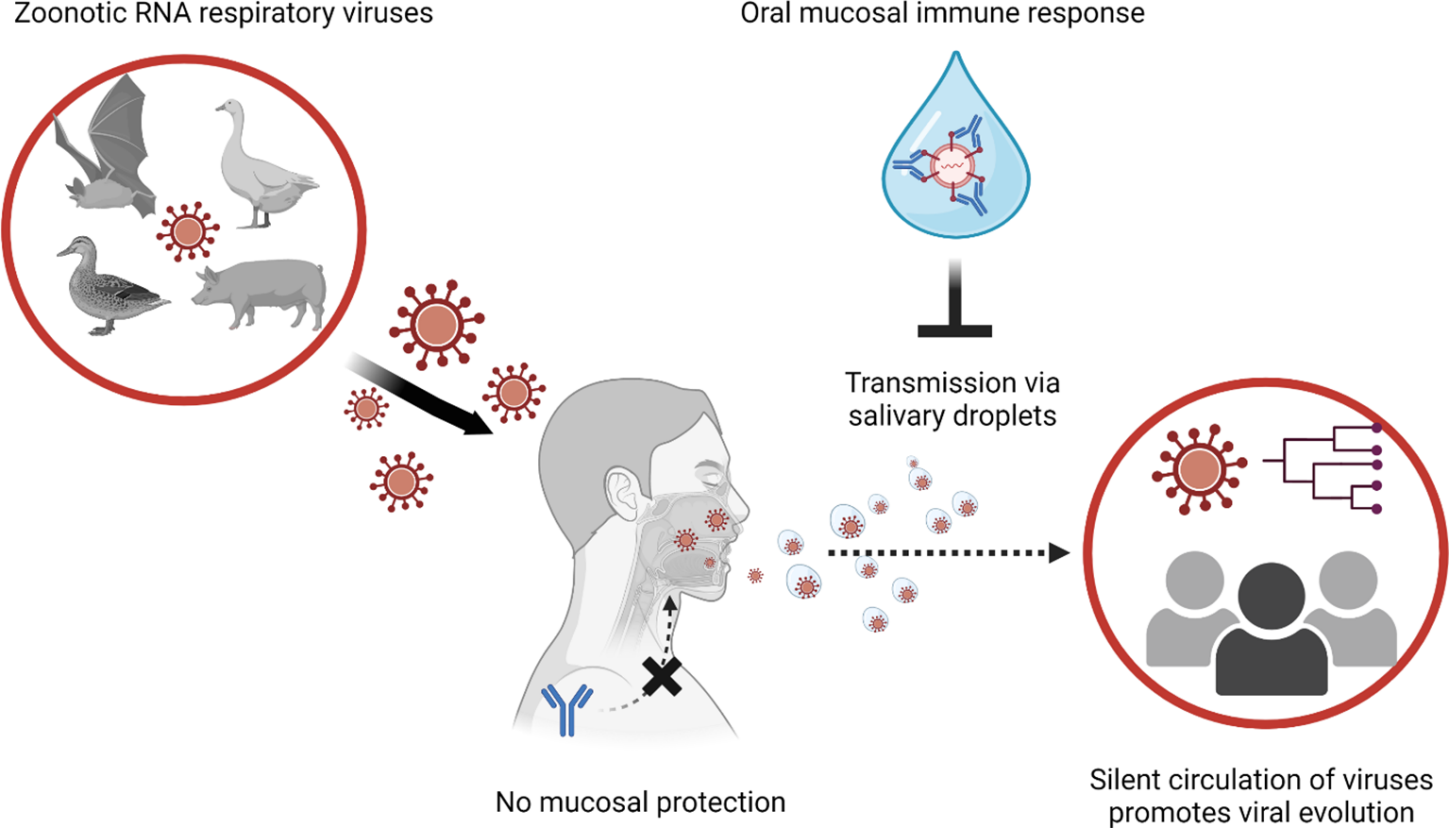
Global transmission viral transmission patterns could be interrupted by mucosal immune responses to manage zoonotic RNA respiratory viruses. Novel viral infections to humans are originated from animals. Especially when zoonotic respiratory RNA viruses gain human-to-human transmission capabilities, the novel infectious agent can explosively spread through the immunologically naive human population. In the case of human populations gaining partial immunity to the virus, systemic antibody response reduces the severity of clinical illness and mortality. However, systemic immune response cannot block the infections/transmission of the viruses at the mucosal surfaces (e.g., upper respiratory tract and oral cavity). In transmission of asymptomatic infection without respiratory symptoms (sneezing and coughing), virus-laden salivary droplets can act as major source of viral transmission. Thus, induction of protective immune response at the oral mucosal surface is instrumental to control transmission of the zoonotic respiratory RNA viruses.

In the case of small virus-laden droplets (<30μm), highly sensitive laser light scattering observations have revealed that loud speech can emit thousands of oral fluid droplets per second (43). In a closed, stagnant air environment, they disappear from the window of view with time constants in the range of 8 to 14 min, corresponding to droplet nuclei of 4μm diameter, or 12 to 21μm droplets prior to dehydration (43). Virus-laden droplets less than 30μm could even spill over conventional facial masks. Spilled RNA virus particles maintain infectivity for hours in the air or on surfaces and infection virus was still detected up to 28 days later (50).The stability of coronaviruses varied between 1 hour to 24 hours depending on the humidity and temperature (51–55). In the case of animal coronavirus porcine enteric diarrhea virus (PEDV), the viral RNA in air was detectable at 16.1 km (56). Actual evidence of airborne transmission has also been demonstrated in *in vitro* and *in vivo* models. Kormuth et al. used humidity-controlled chambers and identified that the 2009 pandemic influenza A (H1N1) virus in suspended aerosols stationary droplets remain infectious for an hour across a wide range of humidities (23-98%) (57). Through a guinea pig model, transmission of influenza A/Panama/2007/1999 (H3N2) (58) virus through the air was measured as efficient as the fomite transmission (58). Collectively, active shedding of respiratory RNA viruses in saliva can be a major source of transmission from asymptomatic carriers lacking respiratory symptoms. Stability of RNA viruses in the air and potential of airborne transmission shows the ease of transmission of the zoonotic respiratory RNA viruses, emphasizing the need for induction of oral immunity (**Figure 1**).

## 3 Induction of oral immunity reduces respiratory viruses spread

The lack of effective measures to prevent entry of viral particles at the mucosal surfaces poses a major challenge in controlling zoonotic respiratory viruses. Vaccination is the most effective strategy to control zoonotic respiratory RNA viral disease, significantly lowering the disease severity and case-fatality rate (59–61). However, current vaccines administered via parenteral route cannot directly stimulate the mucosal immune system (29, 62). Systemic antibodies induced by vaccination provide partial protection to subjects but transportation from blood to mucosal epithelia surface is highly restricted to confer protection at mucosal surfaces (63–65). Instead, vaccinated individuals can carry the viruses without apparent symptoms and serve as asymptomatic carriers (66). As viruses are more attenuated and sheds easier without apparent symptoms, vaccination and symptom-based intervention strategies lose their efficacy and the viruses evolve to more divergent escaping mutants (67). The ultimate strategy to end the current pandemic and prevent future pandemics is to control transmission. Current efforts to control variant viruses are to induce sterilizing immunity, which in turn provides protective immune responses at both mucosal and systemic levels (68, 69). In theory, sterilizing immunity aims to induce neutralizing antibodies at the viral entry site, differentiated from the protective immunity which refers to prevention from symptomatic infections. Sterile immunity prevents the viral transmission, including the asymptomatic and presymptomatic carriers (68, 70). At the phase when the viruses are highly attenuated and asymptomatic transmission lacking respiratory symptoms (e.g., coughing or sneezing) is more frequent, induction of neutralizing IgA response at oral mucosa should be considered (43). While the oral immune system is known to be on the frontline of the gastrointestinal tract (GIT) and respiratory tract, it has been relatively less investigated (71–74). Novel strategies needed to induce oral mucosal immune responses are particularly scarce due to its unique role in preventing entry of external pathogens and hyperactivity to diet or to air exposure.

## 4 The oral mucosal immune system is driven by a unique features

Oral mucosa is the beginning of the GIT and shares anatomic and histologic characteristics with GIT (75–77). In addition to mucus produced by overall GIT, the oral cavity produces saliva (32, 78). The whole saliva is originally generated from serum exudates and supplemented with highly diverse molecules from mucosal cells, immune cells, and microbes (78). Continuous production and swallowing of saliva provide a mechanical clearance of pathogens (78). Also, saliva contains host defense proteins, primarily responsible for both adaptive and innate humoral immune response at oral mucosa (78).

**Figure 2:**
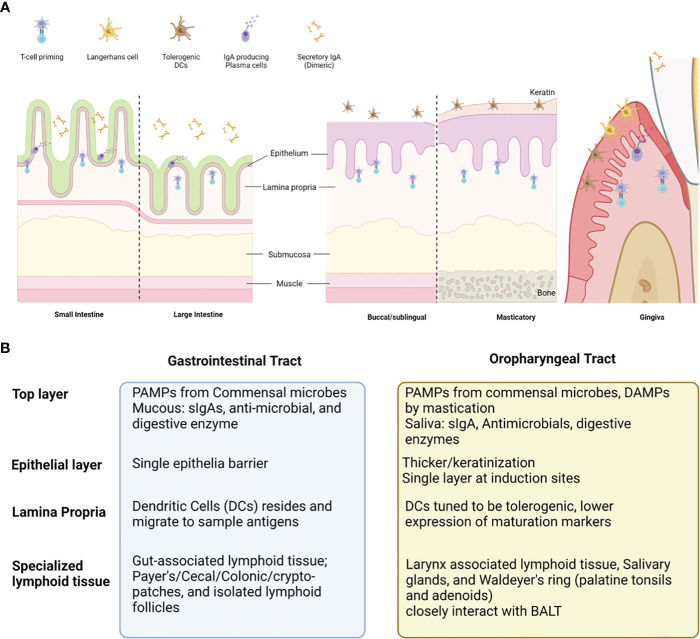
Comparison between oral vs gastrointestinal mucosal tissues and the cell populations contributing to overall immunity. Oral mucosa is the initial compartment for gastrointestinal tract (GIT). Overall structure of oral mucosa is like GIT, consisted with the shares common histologic structure; covered with commensal microbes and saliva filled with diverse antimicrobial, enzymes, and secretory IgAs (sIgAs) **(A)**. The top layer, epithelium, lamina propria, and specialized lymphoid tissues present distinct cells and functions according to the GI versus Oral tract **(B)**. Oral cavity presents unique traits (much thicker epithelial layer, presence of keratin layer, and tolerogenic dendritic cells), which can prevent vaccine antigen delivery and induction of virus-specific immune response at oral mucosal surfaces.

Oral mucosa, like other mucosal tissues, can be divided into three major layers, epithelia, lamina propria, and specialized lymphoid tissues (visual summary in **Figure 2**) (73, 75). The epithelial layer of oral mucosa is stratified squamous epithelium, forming a thicker and denser mechanical barrier than the single layer of GIT epithelia (73, 75). The top portion of the oral epithelial layer forms a level of various levels of keratinization according to the anatomical location (73, 75). Some areas, such as pharynx and junctional epithelium at periodontal space, are non-keratinized and serve as a major point for the innate defense and homeostasis in oral microenvironments (79–81). Lamina propria (LP), a loose connective tissue containing blood and lymphatic vessels under epithelial layers, is a major inductive and effector site for immune cells (79). Steady-state dendritic cells (DCs) reside throughout the lamina propria and often migrate to sample auto-, and foreign antigens derived from commensal microbes, dietary components, mastication damage and pathogens (82). The steady-state DCs in oral tissues are tuned to be tolerogenic to most stimuli from the oral microenvironment, expressing low levels of maturation markers (CD80, CD83, and CD86) (83, 84). In certain conditions, such as invasion of pathogenic microbes, dysbiosis, or damage associated with molecular patterns (DAMPs), the DCs are activated and migrate to lymphoid tissues to induce T activation, such as buccal mucosa, salivary glands, and waldeyer’s ring, is located and serves as major site for activation and expansion of lymphocytes (79). Activated antigen-specific IgA secreting B cells or CD8+ T cells relocate to the effector site, such as the epithelium, LP, and salivary glands, to mediate immune response. But mature DCs also limit T cell activation and promote immune tolerance in specific triggers, such as IL-27, IL-10, vitamin A, or ligands of the aryl hydrocarbon receptor (AhR) (85–88).

As the sIgA can block the viral replication cycle at the initial stage, virus specific sIgAs has been thought to be the most potent target to induce sterilizing immunity at mucosal surfaces (70). In oral mucosa, the sIgA is produced from plasma cells primarily residing in salivary glands and secreted as two monomers linked by a junctional chain via polymeric immunoglobulin receptors (pIgR) at the basolateral membrane of epithelial cells (89–91). In mucosa, the process of class switching to the IgA producing B cell occurs at the lymphoid tissues, such as nasopharyngeal-associated lymphoid tissues (NALT), tear duct associated lymphoid tissue (TALT), and peripheral lymphoid tissues. To elicit antibodies specific to the viral antigen with high affinity, the naive B cells go through the class switch recombination (CSR) by CD40-CD40L ligation in presence of the TGF-β and other co-stimulatory cytokines (IL-4,IL-5, IL-6, IL-10 and IL-21) mediated by CD4+ helper T cells (Th) (91, 92). Meanwhile, naive B cells can activate in response to the continuous stimuli from commensal microbes, metabolites and dietary antigens without involvement of T cells or hypersomatic mutations (93, 94). Two types of antigens have been known to induce the T cell-independent activation (95–97). Type I antigens are typically microbial products (e.g. bacterial LPS or DNA), directly activating B cells through the toll-like receptors on the B cell surface. Type 2 antigens are usually repetitive or highly cross-linked structures found on the surface of encapsulated bacteria, such as polysaccharides or glycolipids. Type II antigens do not have intrinsic activity to stimulate, but accumulation of BCRs and cross-activation of the receptors can activate B lymphocytes, leading to the production of various cytokines, including interleukin-6 (IL-6) and tumor necrosis factor-alpha (TNF-α). Type II antigens can only activate mature B cells. Due to the lack of CSR, sIgAs produced by T cell independent processes present low affinity and low specificity to antigens (98). The source of cytokines involved in T cell independent class-switching is thought to come from subsets of innate immune cells, such as innate lymphoid cells (ILCs) (99). In addition to the sIgA response, commensal microbes are instrumental for the induction and/or tolerance of local immune responses (100–102). Oral mucosa possesses the second largest microbial community after the gut (103, 104). The symbiotic interaction between mucosal epithelial linings and microbes is crucial to maintain steady state of the oral mucosa (100–102).

Microbial colonies serve as primary barriers to inhibit the invasion of external microbes (105). Microbes and their metabolites can also modulate the tone of immune response to constant stimulation by the dietary and inhaled antigens (100–102). Metabolites produced from gut microbiota have been shown to directly influence both inflammatory cells (inflammatory Macs (iMacs), DCs, CD4 T helper (Th)1, CD4Th2, Th17, natural killer (NK) T cells, NK cells and neutrophils) and immuno-suppressive cells (e.g., tolerogenic T cells (T_reg_), regulatory B cells (B_reg_) and innate lymphocytes (ILCs)) (103). Accumulating evidence reveals that the dysbiosis in oral mucosa also contributes to the disease pathogenesis, especially for the respiratory viral infection (103, 106, 107). It is important to note that the oropharynx is the primary site of viral replication and immune induction and major source of the lung microbiome (103, 108). Also, infection with respiratory viruses, such as the SARS-CoV-2, impacts on enrichment of opportunistic pathobionts in the oral cavity (106, 109, 110). A recent cross-sectional study showed that the COVID-19 patients presented a distinctive microbiome profile, a decrease in the alpha-diversity and bacterial species richness in association with symptom severity (103).

The oral cavity maintains homeostatic inflammatory state, created by microflora. The local microflora habituated on the oral cavity is known to be more than 700 species of bacteria, viruses, fungi, and protozoa (111). The main inhabitants of a healthy oral cavity are gram positive and negative cocci and rods, such as Firmicutes, Bacillus, Proteobacteria and Actinomycets (111–113). In a homeostatic state, the microbial community acts as a barrier against colonization of foreign agents and aids differentiation/maturation of the oral immune system (114). For example, constant production of bacterial products and damage associated molecular patterns (DAMPs) constantly recruit and stimulate innate immune cells (eg. neutrophil). Also, bacterial products (LPS, DNA, or polysaccharides) serve as antigen to induce T cell independent low affinity sIgA response during normal state. The sIgAs produced from the healthy state play a pivotal role to prevent overt growth of microbiome. Another important regulator of the microbiome is the fibrin (115). Inflammation triggered by the microbiome results in constant fibrin deposition in oral mucosa. The fibrin activates neutrophil effector functions, harnessing overgrowth of bacteria and activating the plasmin-mediated fibrinolysis. Since the homeostatic inflammation is highly orchestrated by complex interaction among oral mucosa, microbiota, immune cells and clotting factors, dysbiosis and/or tissue damage created by viral infection can significantly impair the oral immune system and promote disease progress from local infection to the systemic illness (103, 115).

## 5 Induction of protective mucosal immune response is challenging: insights on the oral immunology

The oral mucosa is exposed to a variety of environmental insults, including pathogens, allergens, and toxins (77, 116). The oral mucosa is also the first line of defense against these insults, and it is essential that the oral mucosa is able to mount an effective immune response (77, 116). The immune response at mucosal surfaces is mediated by a variety of cells, including dendritic cells, macrophages, neutrophils, and B cells (117). These cells work together to generate an immune response that is specific to the pathogen or allergen that is being encountered (117). While the oral mucosa is constantly stimulated by foreign intakes, the symbiotic interactions among microbes epithelial cells and immune cells can also send signals to the system including clotting factors and microbiome intrusion (83, 84, 115). Due to the complexity, induction of antigen-specific immune response at the oral mucosal surface requires alternative approaches differentiated from conventional parenteral prophylactic or therapeutic strategies. The induction of mucosal immune responses is a complex process that is not fully understood, however, it is known that a number of factors can influence the ability of the oral mucosa to mount an effective immune response (83, 84, 115). These factors include: (i) the presence of pathogens or allergens; (ii) the integrity of the oral mucosa; (ii) the presence of IgA antibodies; (iii) the presence of cytokines; (iv) the presence of regulatory T cells (83, 84, 115, 116). Also, the oral mucosa is home to a variety of commensal bacteria that can interfere with the immune response (83, 84, 115).

The first challenge for inducing mucosal immune response is the multiple mechanical and chemical barriers. Specially, the oral cavity is composed of multiple layers of epithelial cells, most areas covered with keratinized cells, except the inductive sites (pharynx, tonsil, hard/soft palate, buccal-, and sublingual mucosa) (81, 83). Also, the continuous production and swallowing of saliva containing diverse enzymes interferes with stable delivery of vaccine antigens and adjuvants (118). To induce protective immune response, the immunogen needs to overcome such barriers and persist at the site to initiate cascades of immune responses that lead to protection, such as homeostasis and maintenance of health.

The second barrier is to elicit protective immune responses by overcoming oral tolerance without the risk of experiencing hypersensitivity (83, 119, 120). Oral tolerance refers to the process in which the immune system does not respond to orally administered antigens (83, 119). At least two different mechanisms have been identified to mediate development of oral tolerance (83, 119). One mechanism is the induction of regulatory T cells via production of TGF-β but that concomitant retinoic acid signaling boosted this process by mucosal DCs (119). T cell anergy is another possible mechanism induced in high-dose oral tolerance. Anergic T cells are also known to contribute to oral tolerance (83, 119). One method to circumvent oral tolerance could be to apply antigen in another mucosal route, such as intranasal or sublingual route.

Additional considerations relate to inducing protective oral immune response from by influence of commensal microbes and microbiome derived signals (77, 82). Oral cavity maintains homeostatic inflammatory status against commensal microbiota (77, 121, 122). In oral vaccination, depletion of microbiota significantly reduced Th1 and Th17 response to the heat-labile enterotoxin of enterotoxigenic Escherichia coli as adjuvant (LT R192G/L211A) (123). Also, individuals who displayed more diverse gut microbiota tended to exhibit better response to vaccinations (124). In contrast, dysbiosis can result in reduction in vaccine efficacy (125–127). Probiotics have been suggested to enhance IgA and memory T cell response in COVID-19 management (128).

Above issues are the major barrier in developing prophylactic/vaccine strategies to induce oral mucosal immune system and stop the silent spread of the zoonotic respiratory viruses via salivary droplets. Next, we discuss novel approaches targeting influenza, and the SARS-CoV-2 viruses, under clinical trials to prove their efficacy in induction of oral mucosal immunity.

## 6 Novel approaches inducing mucosal immune response specific to the zoonotic respiratory RNA viruses

### 6.1 Delivery system

#### 6.1.1 Direct sensitization of oral mucosa

Is the most efficient route to activate resident immune cells and induce antigen-specific IgA response (129). Novel delivery strategies have been designed to overcome multiple mechanical barriers (e.g., keratinized epithelium, clearance system), proteolytic activity of saliva, and tolerogenic mechanism of oral mucosa. A lipid based delivery system (i.e. liposome, lipid nanoparticles, emulsion and immunostimulatory complexes (ISCOMs)) is a promising vehicle, formulating immunogens in water-immiscible lipid, protecting enzymatic digestion, and enhancing absorption into the mucosal surfaces (130, 131). For COVID-19, the lipid nanoparticle-mRNA format was successfully introduced in an intramuscular injection format. To induce oral mucosal immunity to influenza and COVID-19, the lipid-based delivery system has been tested in *in vivo* studies (132–137).

#### 6.1.2 Polymer-based delivery systems

Can increase the contact time of delivered adjuvant/immunogen, provide stability, and adjunctive effects (138, 139). Polymers can be divided into natural (chitosan, gamma polyglutamic acid, hyaluronic acid, and pullulan) and synthetic (PLGA, Polyethyleneimine, poly-ϵ-caprolactone, PCL, and Polypropylene sulfide). For influenza, the polymer-based vaccines have already been developed and proven their efficacy in animal models for the mucosal influenza vaccine development (140–144). Also, the polymeric-based nanoparticles system is under development for COVID-19 therapeutics and vaccines (145).

#### 6.1.3 Sublingual vaccination

Is also a method of delivering vaccines directly under the tongue, absorbed by the mucous membranes (118, 146). Similar to sublingual vaccination, buccal vaccination is another method of delivering vaccines directly to the mucous membranes in the mouth (118, 146). However, instead of placing the vaccine under the tongue, the vaccine is placed on the inner cheek or buccal mucosa (118, 146). Both sublingual-, and buccal mucosa contains high level of antigen presenting cells, T-, and B-cells and attractive target as vaccine delivery (118, 146, 147). One potential advantage of buccal vaccination over sublingual vaccination is that it may offer more flexibility in terms of vaccine design and formulation. The buccal mucosa has a larger surface area than the sublingual mucosa, which may allow for the delivery of larger doses of the vaccine or the use of more complex formulations (148). There are ongoing research and development efforts to create sublingual vaccines for influenza and coronavirus (including SARS-CoV-2) (118, 149–151). Previous preclinical studies in animals have shown promising results for sublingual vaccines against influenza and coronaviruses, demonstrating the induction of robust immune responses and protection against infection. However, to date, no sublingual vaccine for influenza or coronavirus has been approved for use in humans. Development of the sublingual vaccines for influenza and coronaviruses remains an active area of research, there have been multiple clinical trials (**Table 1**).

**Table 1:** Clinical Trials, Study Phase, and Types of Investigations Related to Oral Mucosa.

1	Phase	Study
*Novel delivery system*		
NCT04334980	Phase I/II	phase 1/2 trial evaluating the safety and immunogenicity of a sublingual influenza vaccine
NCT04625972	Phase I	phase 1 trial evaluating the safety and immunogenicity of a sublingual COVID-19 vaccine
NCT04563702	Phase I	phase 1 trial evaluating the safety and immunogenicity of a buccal COVID-19 vaccine
NCT04644782	Phase II	phase 2 trial evaluating the safety and efficacy of a sublingual COVID-19 vaccine
*Live attenuated/vector vaccine canddiates*		
NCT01982331	Phase II	phase 2 trial evaluating theReactogenicity, Safety and Immunogenicity of a Live Monovalent A/17/California/66/395 (H2N2)Influenza Vaccine
NCT02480101	Phase II	phase 2 trial evaluating theReactogenicity, Safety and Immunogenicity of a Live Monovalent A/17/Anhui/2013/61 (H7N9) Influenza Vaccine
NCT01841918	Phase II	phase 2 trial evaluating the Safety and Immunogenicity of Live Attenuated Influenza H5 Candidate Vaccine Strain A/17/Turkey/Turkey/05/133 (H5N2) in Healthy Thai Volunteers
NCT02229357	non-randominzed open label	non-randomized open label study evaluating thepriming Effects by Pandemic Live Attenuated Influenza Vaccine (LAIV Candidate Vaccine Strain A/17/Turkey/Turkey/05/133 (H5N2)) on the Subsequent Response to Inactivated H5N1 Vaccine in Healthy Thai Volunteers: A Non-Randomized, Open Label Study
NCT03300050	Phase I	phase 1 trial evaluating the Reactogenicity, Safety, and Immunogenicity of a Live Attenuated Universal Influenza Vaccine (cH8/1N1 LAIV) Administered as a Single Priming Dose Followed Three Months Later by a Single Booster Dose of an Inactivated Universal Influenza Vaccine (cH5/1N1 IIV) (Adjuvanted With AS03A or Unadjuvanted) in 18 Through 39 Year-old Healthy Subjects, Contrasted With a Two Dose Schedule of an Inactivated Universal Influenza Vaccine (cH8/1N1 IIV + AS03A Followed Three Months Later by cH5/1N1 IIV + AS03A)
NCT04619628	Phase I	phase 1 trial evaluating the safety and efficacy of a COVI-VAC COVID-19 vaccine
NCT04871737	Phase I	phase 1 trial evaluating the safety and efficacy of a Newcastle disease virus (NDV) vector vaccines expressing the spike protein of SARS-CoV-2
NCT04816019	Phase I	phase 1 trial evaluating the safety and efficacy of a intranasal ChAdOx1 nCoV-19 (AZD1222) COVID-19 vaccine
NCT05007275	Phase I	phase 1 trial evaluating the safety and efficacy of a aerosole ChAdOx1 nCoV-19 (AZD1222) COVID-19 vaccine
NCT04839042	Phase I	phase 1 trial evaluating the safety and efficacy of SC-Ad6-1 COVID-19 vaccine
*Second generation vaccine: Adjuvnat-vaccine complex*		
NCT05385991	Phase I	phase 1 trial evaluating the Safety and Immunogenicity of the ACM-SARS-CoV-2-beta With ACM-CpG Vaccine Candidate (ACM-001), Administered Intramuscularly or Intranasally as a Booster Dose in Healthy Adults Aged 18 to 55 Years, Who Were Previously Vaccinated Against SARS-CoV-2.
*Oral antivirals/antiseptics*		
NCT04405570, NCT04405739	Phase II/III	phase 2/3 trial evaluating the ribonucleoside analogue inhibitor of influenza viruses, MK-4482/EIDD-280 for influenza and SARS-CoV-2 viruses
NCT04405570	Phase Iia	phase 2a trial evaluating the Safety, Tolerability and Efficacy of EIDD-2801 to Eliminate SARS-CoV-2RNA Detection in Persons With COVID-19
NCT04497987	Phase III	phase 3 trial evaluating the Efficacy and Safety of LY3819253 Alone and in Combination With LY3832479 in Preventing SARS-CoV-2 Infection and COVID-19

#### 6.1.4 Microbial display system

Microorganisms, such as virus, bacteria or yeast, can be used as a vaccine delivery system. The microbial display system can leverage their surface proteins as immunostimulants, enhancing immunogenicity of weakly immunogenic vaccine antigens. Also, the *in vitro* cultivation of vehicle microbes enables mass production in a cost-effective way. Bacteria, varial, and fungi have been widely investigated as delivery vehicles. The spore-based system is under clinical trial for the COVID-19 vaccine. (NCT05239923).

### 6.2 Mucosal vaccines

#### 6.2.1 Live attenuated vaccines (LAV)

AV promote direct sensitization of the mucosal surface and have been the most efficacious way to elicit a protective immune response in the oral mucosa (152, 153). Also, live replicating viruses in epithelia stimulates innate and cell-mediated immunity, serving as a self-adjuvant and preserved from the mucosal clearance system. The LAV can be delivered via a variety of routes, including oral, nasal, and rectal (154). Oral delivery of live attenuated vaccines is particularly effective in inducing mucosal immunity (155–158). This attribute has distinct benefits from the parenterally delivered injectable vaccines, including, high efficacy at oral mucosa, ease of administration, and cost effectiveness (28, 159, 160). Also, the LAV is free from the issue of delivery since the vaccine virus attaches to the cellular receptor and is internalized into the mucosal epithelial surface. Activation of innate intracellular signaling pathways during internalization can add self-adjuvanting effects, mainly through the pathogen recognition receptor (PRRs) (161). For seasonal influenza, for example, FluMist has been used over decades as an intranasal spray vaccine (162). Also, there have been multiple live vaccine candidates, such as live attenuated vaccine format and or vector vaccines (163). However, the FluMist could not induce salivary IgA response (164), showing that nasal activation is not always effective.

Multiple live attenuated influenza vaccines have been developed against pandemic influenza strains H5Nx and H7N9 viruses, which is under clinical trials (H2N2: NCT01982331; H7N9: NCT02480101; H5Nx: NCT01841918 and NCT02229357) (165–168). As a next-generation influenza vaccine, a chimeric hemagglutinine-based universal influenza vaccine is also under clinical trial (NCT03300050) (169). Since this novel antigen does not naturally occur, it can avoid the risk of back-mutation. For COVID-19, the COVI-VAC is under phase I clinical trial (NCT04619628).

#### 6.2.2 Vector vaccines

Live vaccines can be designed by using viral vectors, such as Newcastle disease virus (NV), Vestibulo stomatitis virus (VSV), and adenoviruses (170–172). Viral vectors are genetically engineered to express novel antigens, such as the spike protein of the SARS-CoV-2 virus (170–173). As the LAVs, vector viruses attach and replicate directly on the target mucosal tissue, solving the issues of delivery, dosage, and deposition (174). Also, the replication of vector virus triggers the innate and cell-mediated immune system, providing an adjuvant effect for the vaccine antigen (175). A Newcastle disease virus (NDV) vector vaccine expressing the spike protein of SARS-CoV-2 is currently under phase I clinical study (NCT04871737). A replication-competent chimeric VSV-SARS-CoV-2 vaccine candidate by replacing the VSV glycoprotein (G) gene with a coding sequence for the SARS-CoV-2 Spike glycoprotein (S) (VSVΔG-SARS-CoV-2) also has proven efficacy in a hamster model (176). The ChAdOx1 nCoV-19 (AZD1222), developed by AstraZeneca and first approved as an intramuscular vaccine, is now under phase I clinical trial to be applied as intranasal vaccine (NCT04816019) and aerosols (NCT05007275). The SC-Ad6-1 is another adenovirus vector vaccine from Tetherex Pharmaceuticals Corporation also under phase I clinical trial (NCT04839042).

Novel live vaccine candidates under clinical trials are highly expected to be used to complement limitations of current parenteral vaccines. Their efficacy on stopping transmission of viruses is still an emerging topic in the vaccine industry and more accumulated data will be needed.

### 6.3 Mucosal adjuvants

Adjuvants are substances an agent that increases specific immune responses to an antigen (177). Mucosal adjuvants can enhance the immunogenicity of vaccines at the mucosal surface, as evidenced in AS03, MF59, and CpG-ODN (178–180). To enhance the immunogenicity of the vaccines at the mucosal surface, novel adjuvant strategies have been suggested, especially for the influenza vaccine (181–186). Novel approaches apply the microbiome and its byproduct as a source of innate signaling to enhance the antiviral immune response in mucosal surfaces. For example, PMAPs from antibiotic-killed bacteria could enhance antiviral-immune response in intranasal mucosa (187, 188). In a hamster model, Mao et al. applied antibiotic-killed intranasal and oral microbes to induce vaccine-specific nasal IgA and serum IgG responses to influenza and SARS-CoV-2 viruses in a dose-dependent manner (189).

Novel vaccines incorporate adjuvant molecules into vaccine candidates to enhance immunogenicity and delivery system. As a second-generation vaccine, the ACM-SARS-CoV-2-beta ACM-CpG vaccine candidate (ACM-001) is under clinical trial (ClinicalTrials.gov identifier: NCT05385991). The vaccine consists of recombinant Beta spike protein co-administered with synthetic CpG adjuvant. Both components are encapsulated within artificial cell membrane (ACM) polymersomes, synthetic nanovesicles efficiently internalized by antigen-presenting cells, including dendritic cells, enabling targeted delivery of cargo for enhanced immune responses. The ACM vaccine has proven enhanced serum IgG and neutralized response immunogenicity in C57BL/6 mice and Golden Syrian hamsters. In the oral cavity, the ACM-001 vaccination could not reduce the viral peak titer but shortened the viral shedding period (190).

### 6.4 Direct reduction of viral load by using oral antivirals/antiseptics

While the threat of current and future pandemic respiratory viruses is still ongoing, there has not been an effective strategy to induce oral mucosal immunity, especially to novel viruses. Focusing on reducing transmission of the viral spread through saliva droplets, direct administration of antivirals on the oral mucosa can be a temporary alternative strategy to reduce or block viral shedding at oral mucosa. For example, the ribonucleoside analog inhibitor of influenza viruses, MK-4482/EIDD-2801, reported the efficacy for both influenza and SARS-CoV-2 viral infection (currently in phase II/III clinical trials, NCT04405570 and NCT04405739, respectively). In a ferret model, the MK-4482/EIDD-2801 significantly reduced the replication level of the virus at the upper respiratory tract and completely prevented transmission to the contact controls (191). Molnupiravir is also antiviral under clinical trial (NCT04405570), which completely stopped virus shedding from the COVID-19 outpatients by day five after administration via oral route (192) and also active against other RNA viruses, such as influenza, SARS, and MERS. Paxlovid is also an oral antiviral test for COVID-19, reported to shorten the viral shedding period, but it cannot prevent viral infection (193). Antivirals can also be used as prophylaxis to prevent viral infection in the population with high exposure risk. In the case of the influenza virus, antiviral medications (amantadine and neuraminidase inhibitors) are allowed to be used as chemoprophylaxis in people at high risk of influenza complications and people with severe immune deficiencies or receiving immunosuppressive medications (194).

For SARS-CoV-2, repurposing of antivirals as prophylaxis is currently under clinical trial (study NCT04497987). In a stochastic model of early-phase viral infection, the combination of antivirals that block the viral entry and increase viral clearance was estimated to block the small load of viral inoculum (195). Still, the use of antivirals is highly restricted due to their potential side effects and genotoxicity (196). Also, in a primate model, incomplete use of Remdesivir induced a longer duration of viral shedding (197). The combination of Bromelain and Acetylcysteine (BromAc) is under clinical trial to be used as a nebulized form in Healthy volunteers (198). Bromelain, extracted from the pineapple plant (Ananas comosus), contains enzymes that hydrolyze glycosidic bonds in complex carbohydrates and has been shown to remove the spike and hemagglutinin proteins of Semliki Forest virus, Sindbis virus, mouse gastrointestinal coronavirus, hemagglutinating encephalomyelitis virus, and H1N1 influenza viruses (199–201). Acetylcysteine is known to destabilize virion structures by disulfide bridge disruption. The combination use of two molecules unfolds the molecular structures of complex glycoproteins, thus allowing binding to occur because of the high affinity between RBD and ACE2 (198).

To directly reduce/remove viral particles from the oral cavity, antiseptics are also tested under clinical trials and considered to be used. Povidone Iodine has especially shown its efficacy for the oropharyngeal infection (202–204). Since Povidone Iodine has not shown side effects, While hydrogen peroxide can provide an antiseptic effect plus boost the innate immune response by stimulating toll-like receptor 3; the results have been conflicting on the reduction of viral load at the oral mucosal surface (205).

## 7 Future directions and synergistic effects from current vaccines and next-generation vaccines

As COVID-19 pandemic is not considered a “public health emergency”, the risk of the virus spreading and evolving into new variant strains persists. Partial immunity provided by parenteral immunization greatly contributed to reducing the disease severity, but cannot fully stop the spread of the virus, constantly producing novel variant viruses. This review summarized unique characteristics of oral mucosal immunity and discussed strategies currently under clinical trials. Induction of the “sterilizing immunity” is not yet achieved, but there have been remarkable advances in understanding of oral mucosal immune system and vaccine/adjuvants. As a temporary measure to reduce active viral replication at oral mucosa, direct application of antiseptics/antivirals are also considered and under clinical trials. Albeit the limitations, current parenteral vaccines are still the most effective strategy to control pandemic viruses at this present, and emerging mucosal strategies are needed. Even though vaccination provides only partial immunity to mask apparent symptoms and contributes to the silent evolution of the zoonotic respiratory RNA viruses, vaccine-induced immunity reduces the viral load and limits the evolution pool of the viruses, which in turn can hamper transmission. In country-scale analyses on the SARS-CoV-2 genome, diversity of the SARS-CoV-2 virus showed an inverse correlation with the mass vaccine rate (n = 25 countries, mean correlation coefficient = −0.72, S.D. = 0.20) and viruses isolated from vaccinated COVID-19 patients presented significantly lower diversity in known B cell epitopes compared to those from unvaccinated COVID-19 patients (2.3-fold, 95% C.I. 1.4-3.7) (206). Also, pre-existing immunity built by parenteral immunization still provides a booster effect to the mucosal immunization. There have been multiple studies proving the combination of current parenteral vaccinations with mucosal vaccines, providinga synergistic effect on both systemic and mucosal responses (207–209).

Current open questions remaining in the mucosal immune response are 1) What is the sensitive, specific, and reproducible analyte to quantify protective mucosal immune response? 2) what is the complete mechanism involved in oral tolerance and hyperactivity? 3) the most efficient and safe delivery/adjuvant system for the oral mucosa, and 4) the oral microbiome which can contribute elicit protective immune responses. The COVID-19 pandemic has been a unique opportunity to explore diverse strategies against respiratory pathogens. Our current real challenge will be a continuous effort and investment in developing novel strategies to provoke mucosal immunity, especially at oral mucosal sites at a populational scale.

## Author contributions

HJ conceptualized the topic of the article. HJ and MF contributed to build the outline of the paper. HJ and MF wrote the first draft of the paper and MM contributed manuscript revision. HJ and MM created figures, revised by MF. All authors contributed to the article and approved the submitted version.

## References

[1] Kelly H. The classical definition of a pandemic is not elusive. Bull World Health Organ (2011) 89(7):540–1. doi: 10.2471/BLT.11.088815 PMC3127276 21734771

[2] Morens DM Fauci AS. Emerging pandemic diseases: how we got to COVID-19. Cell (2020) 182(5):1077–92. doi: 10.1016/j.cell.2020.08.021 PMC7428724 32846157

[3] Madhav N Oppenheim B Gallivan M Mulembakani P Rubin E Wolfe N. Pandemics: Risks, Impacts, and Mitigation. (Washington, DC: The International Bank for Reconstruction and Development/The World Bank) (2017). 30212163

[4] Morse SS Mazet JAK Woolhouse M Parrish CR Carroll D Karesh WB. Prediction and prevention of the next pandemic zoonosis. Lancet (2012) 380(9857):1956–65. doi: 10.1016/S0140-6736(12)61684-5 PMC3712877 23200504

[5] Batty M. The COVID years: Predictable unpredictability. Environ Plann B: Urban Analytics City Sci (2022) 49(1):3–6. doi: 10.1177/23998083211072588

[6] Pike BL Saylors KE Fair JN Lebreton M Tamoufe U Djoko CF. The origin and prevention of pandemics. Clin Infect Dis (2010) 50(12):1636–40. doi: 10.1086/652860 PMC2874076 20450416

[7] Choudhury PR Saha T Goel S Shah JM Ganjewala D. Cross-species virus transmission and its pandemic potential. Bull Natl Salmon Resour Cent (2022) 46(1):18. doi: 10.1186/s42269-022-00701-7 PMC8787036 35095263

[8] Piret J Boivin G. Pandemics throughout history. Front Microbiol (2020) 11:631736. doi: 10.3389/fmicb.2020.631736 33584597 PMC7874133

[9] Kilbourne ED. Influenza pandemics of the 20th century. Emerg Infect Dis (2006) 12(1):9–14. doi: 10.3201/eid1201.051254 16494710 PMC3291411

[10] Dobson A Foufopoulos J. Emerging infectious pathogens of wildlife. Philos Trans R Soc Lond B Biol Sci (2001) 356(1411):1001–12. doi: 10.1098/rstb.2001.0900 PMC1088495 11516378

[11] Parrish CR Holmes EC Morens DM Park EC Burke DS Calisher CH. Cross-species virus transmission and the emergence of new epidemic diseases. Microbiol Mol Biol Rev (2008) 72(3):457–70. doi: 10.1128/MMBR.00004-08 PMC2546865 18772285

[12] Nova N. Cross-species transmission of coronaviruses in humans and domestic mammals, what are the ecological mechanisms driving transmission, spillover, and disease emergence? Front Public Health (2021) 9:717941. doi: 10.3389/fpubh.2021.717941 34660513 PMC8514784

[13] Klempner MS Shapiro DS. Crossing the species barrier–one small step to man, one giant leap to mankind. N Engl J Med (2004) 350(12):1171–2. doi: 10.1056/NEJMp048039 14985471

[14] Webby R Hoffmann E Webster R. Molecular constraints to interspecies transmission of viral pathogens. Nat Med (2004) 10(12 Suppl):S77–81. doi: 10.1038/nm1151 PMC7095872 15577935

[15] Wolfe ND Dunavan CP Diamond J. Origins of major human infectious diseases. Nature (2007) 447(7142):279–83. doi: 10.1038/nature05775 PMC7095142 17507975

[16] Machalaba CC Loh EH Daszak P Karesh WB. Emerging diseases from animals. In: State of the World 2015: Confronting Hidden Threats to Sustainability. Washington, DC: Island Press/Center for Resource Economics (2015). p. 105–16.

[17] Abdelwhab EM Mettenleiter TC. Zoonotic animal influenza virus and potential mixing vessel hosts. Viruses (2023) 15(4). doi: 10.3390/v15040980 PMC10145017 37112960

[18] Ellwanger JH Chies JAB. Zoonotic spillover: Understanding basic aspects for better prevention. Genet Mol Biol (2021) 44(1 Suppl 1):e20200355. doi: 10.1590/1678-4685-gmb-2020-0355 34096963 PMC8182890

[19] Parrish CR Murcia PR Holmes EC. Influenza virus reservoirs and intermediate hosts: dogs, horses, and new possibilities for influenza virus exposure of humans. J Virol (2015) 89(6):2990–4. doi: 10.1128/JVI.03146-14 PMC4337525 25540375

[20] Shinde V Bridges CB Uyeki TM Shu B Balish A Xu X. Triple-reassortant swine influenza A (H1) in humans in the United States, 2005-2009. N Engl J Med (2009) 360(25):2616–25. doi: 10.1056/NEJMoa0903812 19423871

[21] Garten RJ Davis CT Russell CA Shu B Lindstrom S Balish A. Antigenic and genetic characteristics of swine-origin 2009 A(H1N1) influenza viruses circulating in humans. Science (2009) 325(5937):197–201. doi: 10.1126/science.1176225 19465683 PMC3250984

[22] Woolhouse ME J Adair K Brierley L. RNA viruses: A case study of the biology of emerging infectious diseases. Microbiol Spectr (2013) 1(1). doi: 10.1128/microbiolspec.OH-0001-2012 PMC6157708 26184815

[23] Holmes EC. RNA virus genomics: a world of possibilities. J Clin Invest (2009) 119(9):2488–95. doi: 10.1172/JCI38050 PMC2735898 19729846

[24] Dong J Olano JP McBride JW Walker DH. Emerging pathogens: challenges and successes of molecular diagnostics. J Mol Diagn (2008) 10(3):185–97. doi: 10.2353/jmoldx.2008.070063 PMC2329782 18403608

[25] Carrasco-Hernandez R Jácome R López Vidal Y Ponce de León S. Are RNA viruses candidate agents for the next global pandemic? A review. ILAR J (2017) 58(3):343–58. doi: 10.1093/ilar/ilx026 PMC7108571 28985316

[26] Jones KE Patel NG Levy MA Storeygard A Balk D Gittleman JL. Global trends in emerging infectious diseases. Nature (2008) 451(7181):990–3. doi: 10.1038/nature06536 PMC5960580 18288193

[27] Alvarez-Munoz S Upegui-Porras N Gomez AP Ramirez-Nieto G. Key factors that enable the pandemic potential of RNA viruses and inter-species transmission: A systematic review. Viruses (2021) 13(4). doi: 10.3390/v13040537 PMC8063802 33804942

[28] Pollard AJ Bijker EM. A guide to vaccinology: from basic principles to new developments. Nat Rev Immunol (2021) 21(2):83–100. doi: 10.1038/s41577-020-00479-7 33353987 PMC7754704

[29] Lavelle EC Ward RW. Mucosal vaccines - fortifying the frontiers. Nat Rev Immunol (2022) 22(4):236–50. doi: 10.1038/s41577-021-00583-2 PMC8312369 34312520

[30] Mouro V Fischer A. Dealing with a mucosal viral pandemic: lessons from COVID-19 vaccines. Mucosal Immunol (2022) 15(4):584–94. doi: 10.1038/s41385-022-00517-8 PMC9062288 35505121

[31] Houston S. SARS-CoV-2 mucosal vaccine. Nat Immunol (2023) 24(1):1. doi: 10.1038/s41590-022-01405-w 36596900

[32] Russell MW Mestecky J. Mucosal immunity: The missing link in comprehending SARS-CoV-2 infection and transmission. Front Immunol (2022) 13:957107. doi: 10.3389/fimmu.2022.957107 36059541 PMC9428579

[33] Wang T Wei F Liu J. Emerging role of mucosal vaccine in preventing infection with avian influenza A viruses. Viruses (2020) 12(8). doi: 10.3390/v12080862 PMC7472103 32784697

[34] van de Sandt CE Kreijtz JHCM Rimmelzwaan GF. Evasion of influenza A viruses from innate and adaptive immune responses. Viruses (2012) 4(9):1438–76. doi: 10.3390/v4091438 PMC3499814 23170167

[35] Doherty PC Turner SJ Webby RG Thomas PG. Influenza and the challenge for immunology. Nat Immunol (2006) 7(5):449–55. doi: 10.1038/ni1343 16622432

[36] Read AF Baigent SJ Powers C Kgosana LB Blackwell L Smith LP. Imperfect vaccination can enhance the transmission of highly virulent pathogens. PloS Biol (2015) 13(7):e1002198. doi: 10.1371/journal.pbio.1002198 26214839 PMC4516275

[37] Chakraborty C Sharma AR Bhattacharya M Lee SS. A detailed overview of immune escape, antibody escape, partial vaccine escape of SARS-CoV-2 and their emerging variants with escape mutations. Front Immunol (2022) 13:801522. doi: 10.3389/fimmu.2022.801522 35222380 PMC8863680

[38] Leung NHL. Transmissibility and transmission of respiratory viruses. Nat Rev Microbiol (2021) 19(8):528–45. doi: 10.1038/s41579-021-00535-6 PMC7982882 33753932

[39] Dhand R Li J. Coughs and sneezes: their role in transmission of respiratory viral infections, including SARS-CoV-2. Am J Respir Crit Care Med (2020) 202(5):651–9. doi: 10.1164/rccm.202004-1263PP PMC7462404 32543913

[40] Wang CC Prather KA Sznitman J Jimenez JL Lakdawala SS Tufekci Z. Airborne transmission of respiratory viruses. Science (2021) 373(6558). doi: 10.1126/science.abd9149 PMC8721651 34446582

[41] Raymond JR. The great mask debate: A debate that shouldn’t be a debate at all. WMJ (2020) 119(4):229–39. 33428832

[42] Carrouel F Gadea E Esparcieux A Dimet J Langlois ME Perrier H. Saliva quantification of SARS-CoV-2 in real-time PCR from asymptomatic or mild COVID-19 adults. Front Microbiol (2021) 12:786042. doi: 10.3389/fmicb.2021.786042 35046915 PMC8761670

[43] Stadnytskyi V Bax CE Bax A Anfinrud P. The airborne lifetime of small speech droplets and their potential importance in SARS-CoV-2 transmission. Proc Natl Acad Sci USA (2020) 117(22):11875–7. doi: 10.1073/pnas.2006874117 PMC7275719 32404416

[44] Huang N Pérez P Kato T Mikami Y Okuda K Gilmore RC. SARS-CoV-2 infection of the oral cavity and saliva. Nat Med (2021) 27(5):892–903. doi: 10.1038/s41591-021-01296-8 33767405 PMC8240394

[45] Nicholls JM Bourne AJ Chen H Guan Y Peiris JSM. Sialic acid receptor detection in the human respiratory tract: evidence for widespread distribution of potential binding sites for human and avian influenza viruses. Respir Res (2007) 8(1):73. doi: 10.1186/1465-9921-8-73 17961210 PMC2169242

[46] Baldo V Bertoncello C Cocchio S Fonzo M Pillon P Buja A. The new pandemic influenza A/(H1N1)pdm09 virus: is it really “new”? J Prev Med Hyg (2016) 57(1):E19–22. PMC4910438 27346935

[47] Alkie TN Cox S Embury-Hyatt C Stevens B Pople N Pybus MJ. Characterization of neurotropic HPAI H5N1 viruses with novel genome constellations and mammalian adaptive mutations in free-living mesocarnivores in Canada. Emerg Microbes Infect (2023) 12(1):2186608. doi: 10.1080/22221751.2023.2186608 36880345 PMC10026807

[48] Galar A Catalán P Vesperinas L Miguens I Muñoz I García-Espona A. Use of saliva swab for detection of influenza virus in patients admitted to an emergency department. Microbiol Spectr (2021) 9(1):e0033621. doi: 10.1128/Spectrum.00336-21 34431684 PMC8552598

[49] Tsunetsugu-Yokota Y Ito S Adachi Y Onodera T Kageyama T Takahashi Y. Saliva as a useful tool for evaluating upper mucosal antibody response to influenza. PloS One (2022) 17(2):e0263419. doi: 10.1371/journal.pone.0263419 35130308 PMC8820602

[50] Fukuta M Mao ZQ Morita K Moi ML. Stability and infectivity of SARS-CoV-2 and viral RNA in water, commercial beverages, and bodily fluids. Front Microbiol (2021) 12:667956. doi: 10.3389/fmicb.2021.667956 34025624 PMC8131666

[51] Ijaz MK Brunner AH Sattar SA Nair RC Johnson-Lussenburg CM. Survival characteristics of airborne human coronavirus 229E. J Gen Virol (1985) 66(Pt 12):2743–8. doi: 10.1099/0022-1317-66-12-2743 2999318

[52] van Doremalen N Bushmaker T Munster VJ. Stability of Middle East respiratory syndrome coronavirus (MERS-CoV) under different environmental conditions. Euro Surveill (2013) 18(38). doi: 10.2807/1560-7917.es2013.18.38.20590 24084338

[53] Pyankov OV Bodnev SA Pyankova OG Agranovski IE. Survival of aerosolized coronavirus in the ambient air. J Aerosol Sci (2018) 115:158–63. doi: 10.1016/j.jaerosci.2017.09.009 PMC7094304 32226116

[54] Fears AC Klimstra WB Duprex P Hartman A Weaver SC Plante KC. Comparative dynamic aerosol efficiencies of three emergent coronaviruses and the unusual persistence of SARS-CoV-2 in aerosol suspensions. medRxiv (2020) 1:4–5. doi: 10.1101/2020.04.13.20063784

[55] van Doremalen N Bushmaker T Morris D Holbrook M Gamble A Williamson B. Aerosol and surface stability of SARS-CoV-2 as compared with SARS-CoV-1. N Engl J Med (2020) [Preprint]. doi: 10.1056/NEJMc2004973 PMC7121658 32182409

[56] Alonso C Goede DP Morrison RB Davies PR Rovira A Marthaler DG. Evidence of infectivity of airborne porcine epidemic diarrhea virus and detection of airborne viral RNA at long distances from infected herds. Vet Res (2014) 45(1):73. doi: 10.1186/s13567-014-0073-z 25017790 PMC4347589

[57] Kormuth KA Lin K Prussin AJ 2nd Vejerano EP Tiwari AJ Cox SS. Influenza virus infectivity is retained in aerosols and droplets independent of relative humidity. J Infect Dis (2018) 218(5):739–47. doi: 10.1093/infdis/jiy221 PMC6057527 29878137

[58] Mubareka S Lowen AC Steel J Coates AL García-Sastre A Palese P. Transmission of influenza virus via aerosols and fomites in the Guinea pig model. J Infect Dis (2009) 199(6):858–65. doi: 10.1086/597073 PMC4180291 19434931

[59] Trovato M Sartorius R D’Apice L Manco R De Berardinis P. Viral emerging diseases: challenges in developing vaccination strategies. Front Immunol (2020) 11, 2130. doi: 10.3389/fimmu.2020.02130 33013898 PMC7494754

[60] Huang YZ Kuan CC. Vaccination to reduce severe COVID-19 and mortality in COVID-19 patients: a systematic review and meta-analysis. Eur Rev Med Pharmacol Sci (2022) 26(5):1770–6. doi: 10.26355/eurrev_202203_28248 35302230

[61] Olson SM Newhams MM Halasa NB Feldstein LR Novak T Weiss SL. Vaccine effectiveness against life-threatening influenza illness in US children. Clin Infect Dis (2022) 75(2):230–8. doi: 10.1093/cid/ciab931 35024795

[62] Correa VA Portilho AI De Gaspari E. Vaccines, adjuvants and key factors for mucosal immune response. Immunol (2022) 167(2):124–38. doi: 10.1111/imm.13526 35751397

[63] Hart TK Cook RM Zia-Amirhosseini P Minthorn E Sellers TS Maleeff BE. Preclinical efficacy and safety of mepolizumab (SB-240563), a humanized monoclonal antibody to IL-5, in cynomolgus monkeys. J Allergy Clin Immunol (2001) 108(2):250–7. doi: 10.1067/mai.2001.116576 11496242

[64] Peebles RS Jr Liu MC Lichtenstein LM Hamilton RG. IgA, IgG and IgM quantification in bronchoalveolar lavage fluids from allergic rhinitics, allergic asthmatics, and normal subjects by monoclonal antibody-based immunoenzymetric assays. J Immunol Methods (1995) 179(1):77–86. doi: 10.1016/0022-1759(94)00275-2 7868927

[65] Wu H Pfarr DS Johnson S Brewah YA Woods RM Patel NK. Development of motavizumab, an ultra-potent antibody for the prevention of respiratory syncytial virus infection in the upper and lower respiratory tract. J Mol Biol (2007) 368(3):652–65. doi: 10.1016/j.jmb.2007.02.024 17362988

[66] Acharya CB Schrom J Mitchell AM Coil DA Marquez C Rojas S. Viral load among vaccinated and unvaccinated, asymptomatic and symptomatic persons infected with the SARS-CoV-2 delta variant. Open Forum Infect Dis (2022) 9(5):ofac135. doi: 10.1093/ofid/ofac135 35479304 PMC8992250

[67] Gandhi M Yokoe DS Havlir DV. Asymptomatic transmission, the Achilles’ Heel of current strategies to control Covid-19. N Engl J Med (2020) 382(22):2158–60. doi: 10.1056/NEJMe2009758 PMC7200054 32329972

[68] Focosi D Maggi F Casadevall A. Mucosal vaccines, sterilizing immunity, and the future of SARS-CoV-2 virulence. Viruses (2022) 14(2). doi: 10.3390/v14020187 PMC8878800 35215783

[69] Bouvier NM. The future of influenza vaccines: A historical and clinical perspective. Vaccines (Basel) (2018) 6(3). doi: 10.3390/vaccines6030058 PMC6160951 30200179

[70] Wahl I Wardemann H. Sterilizing immunity: understanding COVID-19. Immunity (2022) 55(12):2231–5. doi: 10.1016/j.immuni.2022.10.017 PMC9595357 36309008

[71] Kurashima Y Kiyono H. Mucosal ecological network of epithelium and immune cells for gut homeostasis and tissue healing. Annu Rev Immunol (2017) 35:119–47. doi: 10.1146/annurev-immunol-051116-052424 28125357

[72] Kurashima Y Yamamoto D Nelson S Uematsu S Ernst PB Nakayama T. Mucosal mesenchymal cells: secondary barrier and peripheral educator for the gut immune system. Front Immunol (2017) 8:1787. doi: 10.3389/fimmu.2017.01787 29321781 PMC5733542

[73] Boyaka PN Fujihashi K. 20 - host defenses at mucosal surfaces. In: Rich RR Fleisher TA Shearer WT Schroeder HW Frew AJ Weyand CM, editors. Clinical Immunology, Fifth Edition. London: Elsevier (2019). p. 285–98.e1.

[74] Kiyono H Yuki Y Nakahashi-Ouchida R Fujihashi K. Mucosal vaccines: wisdom from now and then. Int Immunol (2021) 33(12):767–74. doi: 10.1093/intimm/dxab056 PMC8633596 34436595

[75] Suárez LJ Arboleda S Angelov N Arce RM. Oral versus gastrointestinal mucosal immune niches in homeostasis and allostasis. Front Immunol (2021) 12:705206. doi: 10.3389/fimmu.2021.705206 34290715 PMC8287884

[76] Janeway CA Jr Travers P Walport M Shlomchik MJ. The mucosal immune system. Garland Sci (2001).

[77] Moutsopoulos NM Konkel JE. Tissue-specific immunity at the oral mucosal barrier. Trends Immunol (2018) 39(4):276–87. doi: 10.1016/j.it.2017.08.005 PMC5843496 28923364

[78] Fábián TK Hermann P Beck A Fejérdy P Fábián G. Salivary defense proteins: their network and role in innate and acquired oral immunity. Int J Mol Sci (2012) 13(4):4295–320. doi: 10.3390/ijms13044295 PMC3344215 22605979

[79] Wu RQ Zhang DF Tu E Chen QM Chen W. The mucosal immune system in the oral cavity-an orchestra of T cell diversity. Int J Oral Sci (2014) 6(3):125–32. doi: 10.1038/ijos.2014.48 PMC4170154 25105816

[80] Tsukamoto Y Usui M Yamamoto G Takagi Y Tachikawa T Yamamoto M. Role of the junctional epithelium in periodontal innate defense and homeostasis. J Periodontal Res (2012) 47(6):750–7. doi: 10.1111/j.1600-0765.2012.01490.x 22587460

[81] Brizuela M Winters R. Histology, Oral Mucosa. (Treasure Island, FL: StatPearls Publishing) (2022). 34283481

[82] Meghil MM Cutler CW. Oral microbes and mucosal dendritic cells, “Spark and flame” of local and distant inflammatory diseases. Int J Mol Sci (2020) 21(5). doi: 10.3390/ijms21051643 PMC7084622 32121251

[83] Pelaez-Prestel HF Sanchez-Trincado JL Lafuente EM Reche PA. Immune tolerance in the oral mucosa. Int J Mol Sci (2021) 22(22). doi: 10.3390/ijms222212149 PMC8624028 34830032

[84] Audiger C Rahman MJ Yun TJ Tarbell KV Lesage S. The importance of dendritic cells in maintaining immune tolerance. J Immunol (2017) 198(6):2223–31. doi: 10.4049/jimmunol.1601629 PMC5343761 28264998

[85] Mascanfroni ID Yeste A Vieira SM Burns EJ Patel B Sloma I. IL-27 acts on DCs to suppress the T cell response and autoimmunity by inducing expression of the immunoregulatory molecule CD39. Nat Immunol (2013) 14(10):1054–63. doi: 10.1038/ni.2695 PMC3964005 23995234

[86] Zhou F Broere F Ganguly D. Molecular Mechanisms of Dendritic Cell-Mediated Immune Tolerance and Autoimmunity. (Lausanne, Switzerland: Frontiers Media SA) (2022). 228 p.

[87] Battaglia M Gianfrani C Gregori S Roncarolo MG. IL-10-producing T regulatory type 1 cells and oral tolerance. Ann N Y Acad Sci (2004) 1029:142–53. doi: 10.1196/annals.1309.031 15681753

[88] Quintana FJ Sherr DH. Aryl hydrocarbon receptor control of adaptive immunity. Pharmacol Rev (2013) 65(4):1148–61. doi: 10.1124/pr.113.007823 PMC3799235 23908379

[89] Brandtzaeg P Korsrud FR. Significance of different J chain profiles in human tissues: generation of IgA and IgM with binding site for secretory component is related to the J chain expressing capacity of the total local immunocyte population, including IgG and IgD producing cells, and depends on the clinical state of the tissue. Clin Exp Immunol (1984) 58(3):709–18. PMC1577102 6439452

[90] Kinane DF Lappin DF Koulouri O Buckley A. Humoral immune responses in periodontal disease may have mucosal and systemic immune features. Clin Exp Immunol (1999) 115(3):534–41. doi: 10.1046/j.1365-2249.1999.00819.x PMC1905241 10193430

[91] Boyaka PN. Inducing mucosal igA: A challenge for vaccine adjuvants and delivery systems. J Immunol (2017) 199(1):9–16. doi: 10.4049/jimmunol.1601775 28630108 PMC5719502

[92] Cerutti A. The regulation of IgA class switching. Nat Rev Immunol (2008) 8(6):421–34. doi: 10.1038/nri2322 PMC3062538 18483500

[93] Weller S Braun MC Tan BK Rosenwald A Cordier C Conley ME. Human blood IgM “memory” B cells are circulating splenic marginal zone B cells harboring a prediversified immunoglobulin repertoire. Blood (2004) 104(12):3647–54. doi: 10.1182/blood-2004-01-0346 PMC2590648 15191950

[94] Weller S Faili A Garcia C Braun MC Le Deist FF de Saint Basile GG. CD40-CD40L independent Ig gene hypermutation suggests a second B cell diversification pathway in humans. Proc Natl Acad Sci USA (2001) 98(3):1166–70. doi: 10.1073/pnas.98.3.1166 PMC14726 11158612

[95] Jeurissen A Ceuppens JL Bossuyt X. T lymphocyte dependence of the antibody response to “T lymphocyte independent type 2” antigens. Immunol (2004) 111(1):1–7. doi: 10.1111/j.1365-2567.2003.01775.x PMC1782396 14678191

[96] Fagarasan S Kawamoto S Kanagawa O Suzuki K. Adaptive immune regulation in the gut: T cell-dependent and T cell-independent IgA synthesis. Annu Rev Immunol (2010) 28:243–73. doi: 10.1146/annurev-immunol-030409-101314 20192805

[97] Suzuki K Ha SA Tsuji M Fagarasan S. Intestinal IgA synthesis: a primitive form of adaptive immunity that regulates microbial communities in the gut. Semin Immunol (2007) 19(2):127–35. doi: 10.1016/j.smim.2006.10.001 17161619

[98] Li Y Jin L Chen T. The effects of secretory igA in the mucosal immune system. BioMed Res Int (2020) 2020:2032057. doi: 10.1155/2020/2032057 31998782 PMC6970489

[99] Zheng M Mao K Fang D Li D Lyu J Peng D. B cell residency but not T cell-independent IgA switching in the gut requires innate lymphoid cells. Proc Natl Acad Sci USA (2021) 118(27). doi: 10.1073/pnas.2106754118 PMC8271577 34187897

[100] Zenobia C Herpoldt KL Freire M. Is the oral microbiome a source to enhance mucosal immunity against infectious diseases? NPJ Vaccines (2021) 6(1):80. doi: 10.1038/s41541-021-00341-4 34078913 PMC8172910

[101] Stephen-Victor E Chatila TA. Regulation of oral immune tolerance by the microbiome in food allergy. Curr Opin Immunol (2019) 60:141–7. doi: 10.1016/j.coi.2019.06.001 PMC6800633 31302570

[102] Tordesillas L Berin MC. Mechanisms of oral tolerance. Clin Rev Allergy Immunol (2018) 55(2):107–17. doi: 10.1007/s12016-018-8680-5 PMC6110983 29488131

[103] Soffritti I D’Accolti M Fabbri C Passaro A Manfredini R Zuliani G. Oral microbiome dysbiosis is associated with symptoms severity and local immune/inflammatory response in COVID-19 patients: A cross-sectional study. Front Microbiol (2021) 12:687513. doi: 10.3389/fmicb.2021.687513 34248910 PMC8261071

[104] Caselli E Fabbri C D’Accolti M Soffritti I Bassi C Mazzacane S. Defining the oral microbiome by whole-genome sequencing and resistome analysis: the complexity of the healthy picture. BMC Microbiol (2020) 20(1):120. doi: 10.1186/s12866-020-01801-y 32423437 PMC7236360

[105] König J Wells J Cani PD García-Ródenas CL MacDonald T Mercenier A. Human intestinal barrier function in health and disease. Clin Transl Gastroenterol (2016) 7(10):e196. doi: 10.1038/ctg.2016.54 27763627 PMC5288588

[106] Gupta A Bhanushali S Sanap A Shekatkar M Kharat A Raut C. Oral dysbiosis and its linkage with SARS-CoV-2 infection. Microbiol Res (2022) 261:127055. doi: 10.1016/j.micres.2022.127055 35597076 PMC9065653

[107] Tada A Senpuku H. The impact of oral health on respiratory viral infection. Dent J (2021) 9(4). doi: 10.3390/dj9040043 PMC8069613 33924596

[108] Bassis CM Erb-Downward JR Dickson RP Freeman CM Schmidt TM Young VB. Analysis of the upper respiratory tract microbiotas as the source of the lung and gastric microbiotas in healthy individuals. MBio (2015) 6(2):e00037. doi: 10.1128/mBio.00037-15 25736890 PMC4358017

[109] Yamamoto S Saito M Tamura A Prawisuda D Mizutani T Yotsuyanagi H. The human microbiome and COVID-19: A systematic review. PloS One (2021) 16(6):e0253293. doi: 10.1371/journal.pone.0253293 34161373 PMC8221462

[110] Wu Y Cheng X Jiang G Tang H Ming S Tang L. Author Correction: Altered oral and gut microbiota and its association with SARS-CoV-2 viral load in COVID-19 patients during hospitalization. NPJ Biofilms Microbiomes (2021) 7(1):90. doi: 10.1038/s41522-021-00262-z 34911943 PMC8672330

[111] Lu M Xuan S Wang Z. Oral microbiota: A new view of body health. Food Sci Hum Wellness (2019) 8(1):8–15. doi: 10.1016/j.fshw.2018.12.001

[112] Segata N Haake SK Mannon P Lemon KP Waldron L Gevers D. Composition of the adult digestive tract bacterial microbiome based on seven mouth surfaces, tonsils, throat and stool samples. Genome Biol (2012) 13(6):R42. doi: 10.1186/gb-2012-13-6-r42 22698087 PMC3446314

[113] Mark Welch JL Rossetti BJ Rieken CW Dewhirst FE Borisy GG. Biogeography of a human oral microbiome at the micron scale. Proc Natl Acad Sci USA (2016) 113(6):E791–800. doi: 10.1073/pnas.1522149113 PMC4760785 26811460

[114] Deo PN Deshmukh R. Oral microbiome: Unveiling the fundamentals. J Oral Maxillofac Pathol (2019) 23(1):122–8. doi: 10.4103/jomfp.JOMFP_304_18 PMC6503789 31110428

[115] Silva LM Doyle AD Greenwell-Wild T Dutzan N Tran CL Abusleme L. Fibrin is a critical regulator of neutrophil effector function at the oral mucosal barrier. Science (2021) 374(6575):eabl5450. doi: 10.1126/science.abl5450 34941394 PMC11960105

[116] Şenel S. An overview of physical, microbiological and immune barriers of oral mucosa. Int J Mol Sci (2021) 22(15). doi: 10.3390/ijms22157821 PMC8346143 34360589

[117] Mann ER Li X. Intestinal antigen-presenting cells in mucosal immune homeostasis: crosstalk between dendritic cells, macrophages and B-cells. World J Gastroenterol (2014) 20(29):9653–64. doi: 10.3748/wjg.v20.i29.9653 PMC4123356 25110405

[118] Jahan N Archie SR Shoyaib AA Kabir N Cheung K. Recent approaches for solid dose vaccine delivery. Sci Pharm (2019) 87(4):27. doi: 10.3390/scipharm87040027

[119] Commins SP. Mechanisms of oral tolerance. Pediatr Clin North Am (2015) 62(6):1523–9. doi: 10.1016/j.pcl.2015.07.013 PMC4603531 26456448

[120] Van der Weken H Cox E Devriendt B. Advances in oral subunit vaccine design. Vaccines (Basel) (2020) 9(1). doi: 10.3390/vaccines9010001 PMC7822154 33375151

[121] Kleinstein SE Nelson KE Freire M. Inflammatory networks linking oral microbiome with systemic health and disease. J Dent Res (2020) 99(10):1131–9. doi: 10.1177/0022034520926126 PMC7443998 32459164

[122] Belkaid Y Hand TW. Role of the microbiota in immunity and inflammation. Cell (2014) 157(1):121–41. doi: 10.1016/j.cell.2014.03.011 PMC4056765 24679531

[123] Hall JA Bouladoux N Sun CM Wohlfert EA Blank RB Zhu Q. Commensal DNA limits regulatory T cell conversion and is a natural adjuvant of intestinal immune responses. Immunity (2008) 29(4):637–49. doi: 10.1016/j.immuni.2008.08.009 PMC2712925 18835196

[124] Eloe-Fadrosh EA McArthur MA Seekatz AM Drabek EF Rasko DA Sztein MB. Impact of oral typhoid vaccination on the human gut microbiota and correlations with s. Typhi-specific immunological responses. PloS One (2013) 8(4):e62026. doi: 10.1371/journal.pone.0062026 23637957 PMC3634757

[125] Nadeem S Maurya SK Das DK Khan N Agrewala JN. Gut dysbiosis thwarts the efficacy of vaccine against mycobacterium tuberculosis. Front Immunol (2020) 11:726. doi: 10.3389/fimmu.2020.00726 32508806 PMC7248201

[126] Campillo-Gimenez L Rios-Covian D Rivera-Nieves J Kiyono H Chu H Ernst PB. Microbial-driven immunological memory and its potential role in microbiome editing for the prevention of colorectal cancer. Front Cell Infect Microbiol (2021) 11:752304. doi: 10.3389/fcimb.2021.752304 34869061 PMC8633303

[127] Lynn DJ Pulendran B. The potential of the microbiota to influence vaccine responses. J Leukoc Biol (2018) 103(2):225–31. doi: 10.1189/jlb.5MR0617-216R PMC5921907 28864446

[128] Singh K Rao A. Probiotics: A potential immunomodulator in COVID-19 infection management. Nutr Res (2021) 87:1–12. doi: 10.1016/j.nutres.2020.12.014 33592454 PMC7881295

[129] Huang M Zhang M Zhu H Du X Wang J. Mucosal vaccine delivery: A focus on the breakthrough of specific barriers. Acta Pharm Sin B (2022) 12(9):3456–74. doi: 10.1016/j.apsb.2022.07.002 PMC9259023 35818435

[130] Calderón-Colón X Zhang Y Tiburzi O Wang J Hou S Raimondi G. Design and characterization of lipid nanocarriers for oral delivery of immunotherapeutic peptides. J BioMed Mater Res A (2022) 111(7). doi: 10.1002/jbm.a.37477 36585800

[131] Li M Kaminskas LM Marasini N. Recent advances in nano/microparticle-based oral vaccines. J Pharm Investig (2021) 51(4):425–38. doi: 10.1007/s40005-021-00537-9 PMC8196935 34150345

[132] Sia ZR He X Zhang A Ang JC Shao S Seffouh A. A liposome-displayed hemagglutinin vaccine platform protects mice and ferrets from heterologous influenza virus challenge. Proc Natl Acad Sci USA (2021) 118(22). doi: 10.1073/pnas.2025759118 PMC8179143 34050027

[133] Dhakal S Cheng X Salcido J Renu S Bondra K Lakshmanappa YS. Liposomal nanoparticle-based conserved peptide influenza vaccine and monosodium urate crystal adjuvant elicit protective immune response in pigs. Int J Nanomed (2018) 13:6699–715. doi: 10.2147/IJN.S178809 PMC6205527 30425484

[134] Sato-Kaneko F Yao S Lao FS Shpigelman J Messer K Pu M. A novel synthetic dual agonistic liposomal TLR4/7 adjuvant promotes broad immune responses in an influenza vaccine with minimal reactogenicity. Front Immunol (2020) 11:1207. doi: 10.3389/fimmu.2020.01207 32636840 PMC7318308

[135] Gregoriadis G. Liposomes and mRNA: Two technologies together create a COVID-19 vaccine. Med Drug Discov (2021) 12:100104. doi: 10.1016/j.medidd.2021.100104

[136] Schoenmaker L Witzigmann D Kulkarni JA Verbeke R Kersten G Jiskoot W. mRNA-lipid nanoparticle COVID-19 vaccines: Structure and stability. Int J Pharm (2021) 601:120586. doi: 10.1016/j.ijpharm.2021.120586 33839230 PMC8032477

[137] Liu T Tian Y Zheng A Cui C. Design strategies for and stability of mRNA-lipid nanoparticle COVID-19 vaccines. Polymers (2022) 14(19). doi: 10.3390/polym14194195 PMC9572882 36236141

[138] Chen G Zhao B Ruiz EF Zhang F. Advances in the polymeric delivery of nucleic acid vaccines. Theranostics (2022) 12(9):4081–109. doi: 10.7150/thno.70853 PMC9169366 35673570

[139] Vasquez-Martínez N Guillen D Moreno-Mendieta SA Sanchez S Rodríguez-Sanoja R. The role of mucoadhesion and mucopenetration in the immune response induced by polymer-based mucosal adjuvants. Polymers (2023) 15(7). doi: 10.3390/polym15071615 PMC10097111 37050229

[140] Dhakal S Renu S Ghimire S Shaan Lakshmanappa Y Hogshead BT Feliciano-Ruiz N. Mucosal immunity and protective efficacy of intranasal inactivated influenza vaccine is improved by chitosan nanoparticle delivery in pigs. Front Immunol (2018) 9:934. doi: 10.3389/fimmu.2018.00934 29770135 PMC5940749

[141] Chowdhury MYE Kim TH Uddin MB Kim JH Hewawaduge CY Ferdowshi Z. Mucosal vaccination of conserved sM2, HA2 and cholera toxin subunit A1 (CTA1) fusion protein with poly gamma-glutamate/chitosan nanoparticles (PC NPs) induces protection against divergent influenza subtypes. Vet Microbiol (2017) 201:240–51. doi: 10.1016/j.vetmic.2017.01.020 28284616

[142] Okamoto S Matsuura M Akagi T Akashi M Tanimoto T Ishikawa T. Poly(gamma-glutamic acid) nano-particles combined with mucosal influenza virus hemagglutinin vaccine protects against influenza virus infection in mice. Vaccine (2009) 27(42):5896–905. doi: 10.1016/j.vaccine.2009.07.037 19647814

[143] Singh M Briones M O’Hagan DT. A novel bioadhesive intranasal delivery system for inactivated influenza vaccines. J Control Release (2001) 70(3):267–76. doi: 10.1016/S0168-3659(00)00330-8 11182197

[144] Song L Xiong D Song H Wu L Zhang M Kang X. Mucosal and systemic immune responses to influenza H7N9 antigen HA1-2 co-delivered intranasally with flagellin or polyethyleneimine in mice and chickens. Front Immunol (2017) 8:326. doi: 10.3389/fimmu.2017.00326 28424686 PMC5380672

[145] Charelli LE de Mattos GC de Jesus Sousa-Batista A Pinto JC Balbino TA. Polymeric nanoparticles as therapeutic agents against coronavirus disease. J Nanopart Res (2022) 24(1):12. doi: 10.1007/s11051-022-05396-5 35035277 PMC8747451

[146] Kraan H Vrieling H Czerkinsky C Jiskoot W Kersten G Amorij JP. Buccal and sublingual vaccine delivery. J Control Release (2014) 190, 580–92. doi: 10.1016/j.jconrel.2014.05.060 PMC7114675 24911355

[147] Kweon MN. Sublingual mucosa: A new vaccination route for systemic and mucosal immunity. Cytokine (2011) 54(1):1–5. doi: 10.1016/j.cyto.2010.12.014 21239178

[148] Paris AL Colomb E Verrier B Anjuère F Monge C. Sublingual vaccination and delivery systems. J Control Release (2021) 332:553–62. doi: 10.1016/j.jconrel.2021.03.017 33737202

[149] Shim BS Choi YK Yun CH Lee EG Jeon YS Park SM. Sublingual immunization with M2-based vaccine induces broad protective immunity against influenza. PloS One (2011) 6(11):e27953. doi: 10.1371/journal.pone.0027953 22140491 PMC3227615

[150] Kim H Kim JK Song H Choi J Shim B Kang B. Preliminary study about sublingual administration of bacteria-expressed pandemic H1N1 influenza vaccine in miniature pigs. J Microbiol (2014) 52(9):794–800. doi: 10.1007/s12275-014-4289-4 25079956

[151] Kim Y Park IH Shin J Choi J Jeon C Jeon S. Sublingual dissolving microneedle (SLDMN)-based vaccine for inducing mucosal immunity against SARS-CoV-2. Adv Healthc Mater (2023):e2300889. doi: 10.1002/adhm.202300889 37337388

[152] Nizard M Diniz MO Roussel H Tran T Ferreira LC Badoual C. Mucosal vaccines: novel strategies and applications for the control of pathogens and tumors at mucosal sites. Hum Vaccin Immunother (2014) 10(8):2175–87. doi: 10.4161/hv.29269 PMC4896761 25424921

[153] Shakya AK Chowdhury MYE Tao W Gill HS. Mucosal vaccine delivery: Current state and a pediatric perspective. J Control Release (2016) 240:394–413. doi: 10.1016/j.jconrel.2016.02.014 26860287 PMC5381653

[154] Anggraeni R Ana ID Wihadmadyatami H. Development of mucosal vaccine delivery: an overview on the mucosal vaccines and their adjuvants. Clin Exp Vaccine Res (2022) 11(3):235–48. doi: 10.7774/cevr.2022.11.3.235 PMC9691869 36451668

[155] Johnson S Martinez CI Tedjakusuma SN Peinovich N Dora EG Birch SM. Oral vaccination protects against severe acute respiratory syndrome coronavirus 2 in a Syrian hamster challenge model. J Infect Dis (2022) 225(1):34–41. doi: 10.1093/infdis/jiab561 34758086 PMC8689930

[156] Basak S Kang HJ Lee SH Chu KB Moon EK Quan FS. Influenza vaccine efficacy induced by orally administered recombinant baculoviruses. PloS One (2020) 15(5):e0233520. doi: 10.1371/journal.pone.0233520 32459823 PMC7252623

[157] Mettelman RC Allen EK Thomas PG. Mucosal immune responses to infection and vaccination in the respiratory tract. Immunity (2022) 55(5):749–80. doi: 10.1016/j.immuni.2022.04.013 PMC9087965 35545027

[158] Hellfritzsch M Scherließ R. Mucosal vaccination via the respiratory tract. Pharmaceutics (2019) 11(8). doi: 10.3390/pharmaceutics11080375 PMC6723941 31374959

[159] Minor PD. Live attenuated vaccines: Historical successes and current challenges. Virol (2015) 479-480:379–92. doi: 10.1016/j.virol.2015.03.032 25864107

[160] Belshe RB Edwards KM Vesikari T Black SV Walker RE Hultquist M. Live attenuated versus inactivated influenza vaccine in infants and young children. N Engl J Med (2007) 356(7):685–96. doi: 10.1056/NEJMoa065368 17301299

[161] Pulendran B Ahmed R. Immunological mechanisms of vaccination. Nat Immunol (2011) 12(6):509–17. doi: 10.1038/ni.2039 PMC3253344 21739679

[162] Shannon I White CL Nayak JL. Understanding immunity in children vaccinated with live attenuated influenza vaccine. J Pediatr Infect Dis Soc (2020) 9(Supplement_1):S10–4. doi: 10.1093/jpids/piz083 31848606

[163] Alu A Chen L Lei H Wei Y Tian X Wei X. Intranasal COVID-19 vaccines: From bench to bed. EBioMed (2022) 76:103841. doi: 10.1016/j.ebiom.2022.103841 PMC8785603 35085851

[164] Ponzio TA Sanders JW. The salivary gland as a target for enhancing immunization response. Trop Dis Travel Med Vaccines (2017) 3:4. doi: 10.1186/s40794-017-0047-z 28883974 PMC5531011

[165] Ortiz JR Spearman PW Goepfert PA Cross K Buddy Creech C Chen WH. Safety and immunogenicity of monovalent H7N9 influenza vaccine with AS03 adjuvant given sequentially or simultaneously with a seasonal influenza vaccine: A randomized clinical trial. Vaccine (2022) 40(23):3253–62. doi: 10.1016/j.vaccine.2022.03.055 PMC9897630 35465983

[166] Pitisuttithum P Boonnak K Chamnanchanunt S Puthavathana P Luvira V Lerdsamran H. Safety and immunogenicity of a live attenuated influenza H5 candidate vaccine strain A/17/Turkey/Turkey/05/133 H5N2 and its priming effects for potential pre-pandemic use: a randomised, double-blind, placebo-controlled trial. Lancet Infect Dis (2017) 17(8):833–42. doi: 10.1016/S1473-3099(17)30240-2 PMC5522535 28533093

[167] Isakova-Sivak I Stukova M Erofeeva M Naykhin A Donina S Petukhova G. H2N2 live attenuated influenza vaccine is safe and immunogenic for healthy adult volunteers. Hum Vaccin Immunother (2015) 11(4):970–82. doi: 10.1080/21645515.2015.1010859 PMC4514355 25831405

[168] Liebowitz D Gottlieb K Kolhatkar NS Garg SJ Asher JM Nazareno J. Efficacy, immunogenicity, and safety of an oral influenza vaccine: a placebo-controlled and active-controlled phase 2 human challenge study. Lancet Infect Dis (2020) 20(4):435–44. doi: 10.1016/S1473-3099(19)30584-5 31978354

[169] Nachbagauer R Feser J Naficy A Bernstein DI Guptill J Walter EB. A chimeric hemagglutinin-based universal influenza virus vaccine approach induces broad and long-lasting immunity in a randomized, placebo-controlled phase I trial. Nat Med (2021) 27(1):106–14. doi: 10.1038/s41591-020-1118-7 33288923

[170] García-Sastre A. Mucosal delivery of RNA vaccines by Newcastle disease virus vectors. Curr Res Immunol (2022) 3:234–8. doi: 10.1016/j.crimmu.2022.10.001 PMC9552541 36245642

[171] Fathi A Dahlke C Addo MM. Recombinant vesicular stomatitis virus vector vaccines for WHO blueprint priority pathogens. Hum Vaccin Immunother (2019) 15(10):2269–85. doi: 10.1080/21645515.2019.1649532 PMC6816421 31368826

[172] Sakurai F Tachibana M Mizuguchi H. Adenovirus vector-based vaccine for infectious diseases. Drug Metab Pharmacokinet (2022) 42:100432. doi: 10.1016/j.dmpk.2021.100432 34974335 PMC8585960

[173] Sun W Leist SR McCroskery S Liu Y Slamanig S Oliva J. Newcastle disease virus (NDV) expressing the spike protein of SARS-CoV-2 as a live virus vaccine candidate. EBioMed (2020) 62:103132. doi: 10.1016/j.ebiom.2020.103132 PMC7679520 33232870

[174] Travieso T Li J Mahesh S Mello JDFRE Blasi M. The use of viral vectors in vaccine development. NPJ Vaccines (2022) 7(1):75. doi: 10.1038/s41541-022-00503-y 35787629 PMC9253346

[175] Chiale C Marchese AM Furuya Y Robek MD. Virus-based vaccine vectors with distinct replication mechanisms differentially infect and activate dendritic cells. NPJ Vaccines (2021) 6(1):138. doi: 10.1038/s41541-021-00400-w 34811393 PMC8608815

[176] Espeseth AS Yuan M Citron M Reiserova L Morrow G Wilson A. Preclinical immunogenicity and efficacy of a candidate COVID-19 vaccine based on a vesicular stomatitis virus-SARS-CoV-2 chimera. EBioMed (2022) 82:104203. doi: 10.1016/j.ebiom.2022.104203 PMC9338221 35915046

[177] Pulendran B Arunachalam P S O’Hagan DT. Emerging concepts in the science of vaccine adjuvants. Nat Rev Drug Discov (2021) 20(6):454–75. doi: 10.1038/s41573-021-00163-y PMC8023785 33824489

[178] New RRC. Formulation technologies for oral vaccines. Clin Exp Immunol (2019) 198(2):153–69. doi: 10.1111/cei.13352 PMC6797897 31318446

[179] Wilkins AL Kazmin D Napolitani G Clutterbuck EA Pulendran B Siegrist CA. AS03- and MF59-adjuvanted influenza vaccines in children. Front Immunol (2017) 8:1760. doi: 10.3389/fimmu.2017.01760 29326687 PMC5733358

[180] Iho S Maeyama JI Suzuki F. CpG oligodeoxynucleotides as mucosal adjuvants. Hum Vaccin Immunother (2015) 11(3):755–60. doi: 10.1080/21645515.2014.1004033 PMC4514178 25751765

[181] Ichinohe T Watanabe I Ito S Fujii H Moriyama M Tamura SI. Synthetic double-stranded RNA poly(I:C) combined with mucosal vaccine protects against influenza virus infection. J Virol (2005) 79(5):2910–9. doi: 10.1128/JVI.79.5.2910-2919.2005 PMC548446 15709010

[182] Spinner JL Oberoi HS Yorgensen YM Poirier DS Burkhart DJ Plante M. Methylglycol chitosan and a synthetic TLR4 agonist enhance immune responses to influenza vaccine administered sublingually. Vaccine (2015) 33(43):5845–53. doi: 10.1016/j.vaccine.2015.08.086 PMC4609623 26392012

[183] Ainai A Ichinohe T Tamura SI Kurata T Sata T Tashiro M. Zymosan enhances the mucosal adjuvant activity of poly(I:C) in a nasal influenza vaccine. J Med Virol (2010) 82(3):476–84. doi: 10.1002/jmv.21694 20087927

[184] Skountzou I Martin M del P Wang B Ye L Koutsonanos D Weldon W. Salmonella flagellins are potent adjuvants for intranasally administered whole inactivated influenza vaccine. Vaccine (2010) 28(24):4103–12. doi: 10.1016/j.vaccine.2009.07.058 PMC3187848 19654062

[185] Sjölander S Drane D Davis R Beezum L Pearse M Cox J. Intranasal immunisation with influenza-ISCOM induces strong mucosal as well as systemic antibody and cytotoxic T-lymphocyte responses. Vaccine (2001) 19(28-29):4072–80. doi: 10.1016/S0264-410X(01)00110-4 11427284

[186] Bracci L Canini I Puzelli S Sestili P Venditti M Spada M. Type I IFN is a powerful mucosal adjuvant for a selective intranasal vaccination against influenza virus in mice and affects antigen capture at mucosal level. Vaccine (2005) 23(23):2994–3004. doi: 10.1016/j.vaccine.2004.12.006 15811645

[187] Matsuo K Yoshikawa T Asanuma H Iwasaki T Hagiwara Y Chen Z. Induction of innate immunity by nasal influenza vaccine administered in combination with an adjuvant (cholera toxin). Vaccine (2000) 18(24):2713–22. doi: 10.1016/S0264-410X(00)00055-4 10781859

[188] Gopinath S Kim MV Rakib T Wong PW van Zandt M Barry NA. Topical application of aminoglycoside antibiotics enhances host resistance to viral infections in a microbiota-independent manner. Nat Microbiol (2018) 3(5):611–21. doi: 10.1038/s41564-018-0138-2 PMC5918160 29632368

[189] Nagai M Moriyama M Ichinohe T. Oral bacteria combined with an intranasal vaccine protect from influenza A virus and SARS-CoV-2 infection. MBio (2021) 12(4):e0159821. doi: 10.1128/mBio.01598-21 34399617 PMC8406166

[190] Lam JH Shivhare D Chia TW Chew SL Sinsinbar G Aw TY. Artificial cell membrane polymersome-based intranasal beta spike formulation as a second generation Covid-19 vaccine. ACS Nano (2022) 16(10):16757–75. doi: 10.1021/acsnano.2c06350 36223228

[191] Cox RM Wolf JD Plemper RK. Therapeutically administered ribonucleoside analogue MK-4482/EIDD-2801 blocks SARS-CoV-2 transmission in ferrets. Nat Microbiol (2021) 6(1):11–8. doi: 10.1038/s41564-020-00835-2 PMC7755744 33273742

[192] Fischer WA 2nd Eron JJ Jr Holman W Cohen MS Fang L Szewczyk LJ. A phase 2a clinical trial of molnupiravir in patients with COVID-19 shows accelerated SARS-CoV-2 RNA clearance and elimination of infectious virus. Sci Transl Med (2022) 14(628):eabl7430. doi: 10.1126/scitranslmed.abl7430 34941423 PMC10763622

[193] Zhong W Jiang X Yang X Feng T Duan Z Wang W. The efficacy of paxlovid in elderly patients infected with SARS-CoV-2 omicron variants: Results of a non-randomized clinical trial. Front Med (2022) 9, 980002. doi: 10.3389/fmed.2022.980002 PMC9485497 36148451

[194] CDC. Influenza antiviral medications: Summary for clinicians. Centers for Disease Control and Prevention, National Center for Immunization and Respiratory Diseases (NCIRD, online source). (2022). Available at: https://www.cdc.gov/flu/professionals/antivirals/summary-clinicians.htm.

[195] Czuppon P Débarre F Gonçalves A Tenaillon O Perelson AS Guedj J. Success of prophylactic antiviral therapy for SARS-CoV-2: Predicted critical efficacies and impact of different drug-specific mechanisms of action. PloS Comput Biol (2021) 17(3):e1008752. doi: 10.1371/journal.pcbi.1008752 33647008 PMC7951973

[196] Zhou S Hill CS Sarkar S Tse LV Woodburn BMD SChinazi RF. β-d-N4-hydroxycytidine inhibits SARS-CoV-2 through lethal mutagenesis but is also mutagenic to mammalian cells. J Infect Dis (2021) 224(3):415–9. doi: 10.1093/infdis/jiab247 PMC8136050 33961695

[197] Kim KS Iwanami S Oda T Fujita Y Kuba K Miyazaki T. Incomplete antiviral treatment may induce longer durations of viral shedding during SARS-CoV-2 infection. Life Sci Alliance (2021) 4(10). doi: 10.26508/lsa.202101049 PMC8340032 34344719

[198] Akhter J Quéromès G Pillai K Kepenekian V Badar S Mekkawy AH. The combination of bromelain and acetylcysteine (BromAc) synergistically inactivates SARS-CoV-2. Viruses (2021) 13(3). doi: 10.3390/v13030425 PMC7999995 33800932

[199] Amini A Masoumi-Moghaddam S Morris DL. Utility of Bromelain and N-Acetylcysteine in Treatment of Peritoneal Dissemination of Gastrointestinal Mucin-Producing Malignancies. (Kapandriti, Greece: Springer) (2016). 229 p. 27272863

[200] Schlegel A Schaller J Jentsch P Kempf C. Semliki Forest virus core protein fragmentation: its possible role in nucleocapsid disassembly. Biosci Rep (1993) 13(6):333–47. doi: 10.1007/BF01150478 8204803

[201] Greig AS Bouillant AM. Binding effects of concanavalin A on a coronavirus. Can J Comp Med (1977) 41(1):122–6. PMC1277703 832184

[202] Lim NA Teng O Ng CYH Bao LXY Tambyah PA Quek AML. Repurposing povidone-iodine to reduce the risk of SARS-CoV-2 infection and transmission: a narrative review. Ann Med (2022) 54(1):1488–99. doi: 10.1080/07853890.2022.2076902 PMC9132411 35594333

[203] Naqvi SHS Citardi MJ Cattano D Ostrosky-Zeichner L Knackstedt MI Karni RJ. Povidone-iodine solution as SARS-CoV-2 prophylaxis for procedures of the upper aerodigestive tract a theoretical framework. J Otolaryngol Head Neck Surg (2020) 49(1):77. doi: 10.1186/s40463-020-00474-x 33109269 PMC7590913

[204] Kanagalingam J Feliciano R Hah JH Labib H Le TA Lin JC. Practical use of povidone-iodine antiseptic in the maintenance of oral health and in the prevention and treatment of common oropharyngeal infections. Int J Clin Pract (2015) 69(11):1247–56. doi: 10.1111/ijcp.12707 PMC6767541 26249761

[205] Mateos-Moreno MV Mira A Ausina-Márquez V Ferrer MD. Oral antiseptics against coronavirus: *in-vitro* and clinical evidence. J Hosp Infect (2021) 113:30–43. doi: 10.1016/j.jhin.2021.04.004 33865974 PMC8046704

[206] Mueller S. Challenges and Opportunities of mRNA Vaccines Against SARS-CoV-2: A Multidisciplinary Perspective. (New York, USA: Springer Nature) (2023). 439 p.

[207] Mao T Israelow B Peña-Hernández MA Suberi A Zhou L Luyten S. Unadjuvanted intranasal spike vaccine elicits protective mucosal immunity against sarbecoviruses. Science (2022) 378(6622):eabo2523. doi: 10.1126/science.abo2523 36302057 PMC9798903

[208] Yang Z Zhao Q Gao YA Zhang W. Combined oral and intravenous immunization stimulates strong igA responses in both systemic and mucosal compartments. PloS One (2016) 11(12):e0168037. doi: 10.1371/journal.pone.0168037 27936222 PMC5148103

[209] Tsunetsugu-Yokota Y Ishige M Murakami M. Oral attenuated Salmonella enterica serovar Typhimurium vaccine expressing codon-optimized HIV type 1 Gag enhanced intestinal immunity in mice. AIDS Res Hum Retroviruses (2007) 23(2):278–86. doi: 10.1089/aid.2006.0098 17331034

